# YAP modulates TGF-β1-induced simultaneous apoptosis and EMT through upregulation of the EGF receptor

**DOI:** 10.1038/srep45523

**Published:** 2017-04-20

**Authors:** Yi Liu, Kai He, Ying Hu, Xiaojie Guo, Dongmei Wang, Weiwei Shi, Jingsong Li, Jianguo Song

**Affiliations:** 1 State Key Laboratory of Cell Biology, CAS Center for Excellence in Molecular Cell Science, Innovation Center for Cell Signaling Network, Shanghai Institute of Biochemistry and Cell Biology, Chinese Academy of Sciences, Shanghai, 200031, China

## Abstract

YAP is a transcriptional co-regulator that plays important roles in various patho-physiological processes, including the survival and death of cells. However, the effect of YAP on apoptosis and EMT, simultaneously mediated by TGF-β1, is not known. In this study, we demonstrate that YAP can modulate cell fate of apoptosis versus EMT by acting as a surviving factor. Overexpression of YAP in mouse mammary epithelial (NMuMG) cells suppressed TGF-β1-induced apoptosis, which shifted the cellular response predominantly toward EMT. In contrast, knockdown of YAP induced spontaneous apoptosis and enhanced TGF-β1-induced apoptosis, leading to a sharp decrease in the proportion of surviving cells that underwent EMT. These data suggest that YAP is an essential factor for modulating cellular responses to TGF-β1. Further investigation showed that YAP could regulate the expression level and activation of EGFR. Knockdown or inhibition of EGFR abolished the suppressive effect of YAP on apoptosis, whereas activation of EGFR by EGF significantly reduced apoptosis caused by the knockdown of YAP. The results indicate that EGFR and its activation are critical for YAP-mediated suppression of TGF-β1-induced apoptosis. This study provides a new understanding of the regulatory mechanism underlying the determination of cell fate in response to TGF-β1-mediated simultaneous apoptosis and EMT.

Apoptosis is widely known as a basic biological event. It is a strictly programmed process for removing superfluous, aged, or damaged cells. Apoptosis is characterized by cell shrinkage, membrane blebbing, and nuclear condensation and fragmentation. During embryonic development, apoptosis is an essential event required for successful organogenesis. In adult tissues, apoptosis counterbalances cell proliferation to maintain cell numbers and tissue homeostasis^1^,^2^,^3^. Dysregulation of apoptosis can result in abnormal cell growth and the relevant diseases. A large number of stimuli, both natural and non-natural, have been found to regulate apoptosis. For instance, TGF-β1, TNF-α, ROS, p53, and various chemotherapy drugs^4^,^5^,^6^,^7^ were found to be potent inducers of apoptosis.

TGF-β1 is a pleiotropic cytokine, which is implicated in the control of diverse cellular processes, such as cell cycle state, cell differentiation, chemotaxis and migration^8^. In physiological processes, TGF-β1 contributes to cellular homeostasis, embryonic development, immune response, wound healing, and angiogenesis^9^. However, in pathological processes, TGF-β1 signaling has been linked with progression of diseases, such as organ fibrosis and cancers^10^,^11^. It is known that TGF-β1 can function as pro-tumor factor by increasing cell motility, invasion, and angiogenesis. The role of TGF-β1 depends on cell type, cellular context, cell state, and the cell environment. Although TGF-β1 is a multifunctional molecule, a singular response to TGF-β1 treatment was found in most cell types. However, TGF-β1 can simultaneously induce both apoptosis and EMT, two fundamentally different cell fates, in certain cells. For example, mouse hepatocytes^12^ and mammary gland epithelial cells^13^ respond simultaneously to TGF-β1 treatment with apoptosis and EMT. In other words, in a population of the exact same type of cells, some undergo apoptosis and some undergo EMT in response to TGF-β1. It has been shown previously that TGF-β1-induced apoptosis and EMT were cell cycle dependent events^14^. Cells synchronized at G1/S phase primarily undergo EMT, while cells synchronized at G2/M phase undergo apoptosis. Strikingly, TGF-β1-mediated growth arrest has been found to be closely associated with its induction of EMT in non-cancer cells. Although TGF-β1 can induce both apoptosis and EMT in certain non-cancer cells, most types of cancer cells of epithelial origin escaped the apoptotic induction effect of TGF-β1. Because of the high heterogeneity of cancer cells, whether TGF-β1 is able to induce EMT in a type of cancer cell depends on the cellular context and the cell plasticity. Further exploration of the molecules implicated in inhibition of TGF-β1-induced apoptosis in normal cells is important for achieving a better understanding of the changes that convert cells from an apoptosis-inducible to an apoptosis-resistant state.

Interactions between Hippo and TGF-β signaling pathways has been shown in recent years^15^,^16^,^17^,^18^. YAP is a key component of the Hippo pathway, which functions as a transcriptional co-activator. YAP has been shown to play important roles in regulating proliferation, survival, self-renewal and organ size^19^. YAP and other core Hippo pathway components were initially identified in Drosophila and are highly conserved in mammals^20^. The core kinase cassette in the Hippo pathway consists of a series of serine/threonine kinases of MST and LATS families^21^. When the Hippo pathway is on, activated MSTs/LATSs induce the phosphorylation of serine 127 in YAP, which promotes 14-3-3 binding and leads to cytoplasmic retention of YAP^22^,^23^. In the absence of activated Hippo, YAP translocates into cell nucleus and promotes downstream transcription networks by interacting with the TEAD family of transcription factors^24^,^25^.

In mammalian species, functional studies of YAP have been primarily performed in tumor cells. YAP has been shown to play a role in promoting tumorigenesis, cell proliferation, resistance of apoptosis, anchorage-independent growth *in vitro*, migration, and metastasis of tumor cells^26^,^27^. Overexpression of YAP inhibits apoptosis induced by cisplatin, paclitaxel, UV irradiation, and ionizing irradiation^28^,^29^,^30^,^31^. Increased YAP expression or its nuclear localization has been observed in a variety of human cancers^27^. However, there are also reports showing that YAP functions as a pro-apoptotic effector in mammalian cells. YAP overexpression increases p73-mediated apoptosis upon DNA damage^32^,^33^. The pro-apoptotic role of YAP is thought to be dependent on different cellular contexts and related downstream molecules^34^. Moreover, compared to non-natural inducers in cancer cells, little is known about the effect of YAP on apoptosis induced by physiological stimuli, particularly in normal cells. In addition, in apoptosis and EMT simultaneously induced by TGF-β1, the effect of YAP on these two fundamentally different cell fates has not been investigated.

In this study, we examined whether YAP has a regulatory effect on TGF-β1-induced apoptosis and EMT. We found that YAP overexpression can inhibit TGF-β1-induced apoptosis. Although overexpression of YAP alone cannot induce EMT directly, it can increase the number of cells undergoing EMT. YAP knockdown induced apoptosis and dramatically increased the apoptotic response of cells to TGF-β1 treatment, which significantly reduced the portion of survival cells that underwent EMT. In addition, we determined that increased EGFR levels and its activation are involved in YAP-mediated suppression of TGF-β1-induced apoptosis. The data presented suggest that the YAP expression level is implicated in modulating the cellular fates of apoptosis and EMT in response to TGF-β1 stimulation.

## Results

### Increases in YAP expression in TGFβ1-induced apoptosis and EMT

**Figure 1:**
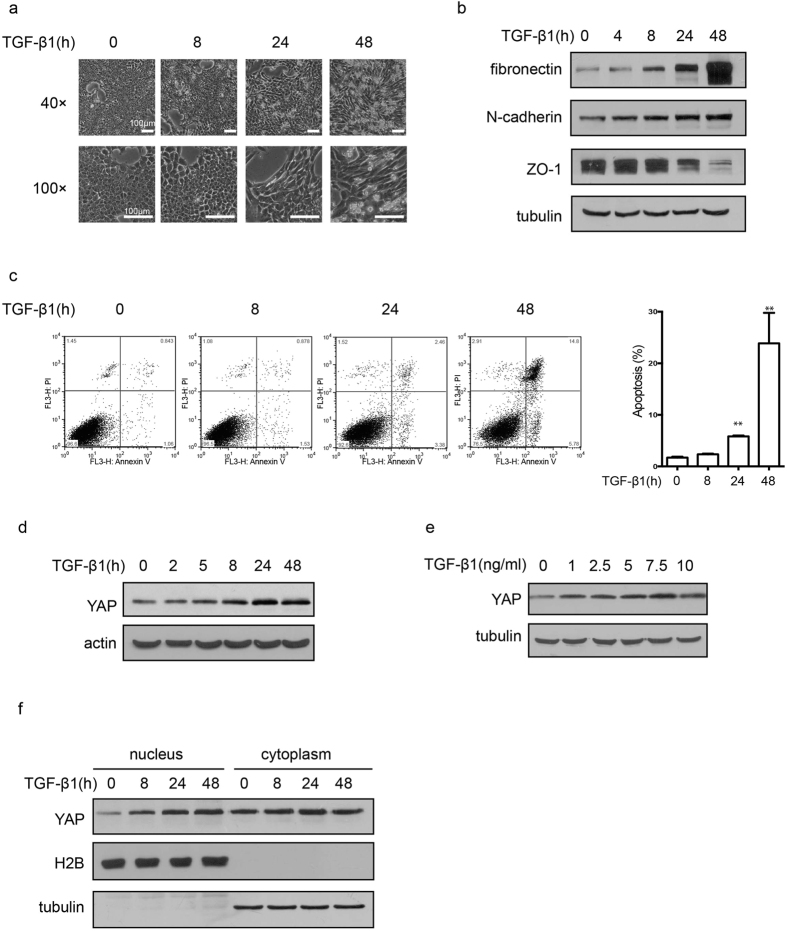
Increased YAP levels in TGF-β1-induced apoptosis and EMT. (**a**) Cell morphological changes were detected in NMuMG cells after TGF-β1 (10 ng/mL) treatment for the indicated time. (**b**) The effect of TGF-β1 (10 ng/mL, 48 h) on the protein level of EMT markers was detected by immune blotting (Epithelial marker: ZO-1. Mesenchymal markers: fibronectin and N-cadherin). (**c**) The effect of TGF-β1 (10 ng/mL) on apoptosis was detected by Annexin V/PI double staining and analyzed by flow cytometry. The representative images (left) and statistical data (right) were shown. The data are the means ± SD of three independent experiments. (**d**) Time-dependent effect of increased YAP expression level was detected by immunoblotting after TGF-β1 (10 ng/mL) treatment in NMuMG cells. (**e**) The dosage-effect of TGF-β1 on the expression level of YAP. Cells were treated with different concentration of TGF-β1 for 48 h. (**f**) The cytoplasmic and nuclear YAP levels were examined after TGF-β1 (10 ng/mL) treatment for the indicated time.

We first examined the relationship between YAP expression level and TGF-β1 signaling and its association with TGF-β1-mediated apoptosis and EMT in normal mouse mammary epithelial cells (NMuMG). Compared to cancer cell lines that escape the apoptosis-inducing effect of TGF-β1, NMuMG cells simultaneously undergo both apoptosis and EMT, which can be directly observed by cell morphology (Fig. 1a). EMT and apoptosis were also determined by changes in epithelial and mesenchymal marker protein levels and by Annexin V/PI double staining, respectively. As shown, both EMT (Fig. 1b) and apoptosis (Fig. 1c) exhibited time-dependent effects. Additionally, a dose-dependent effect on apoptosis was also observed (Supplementary Fig. S1). Interestingly, a moderate time-dependent increase in the YAP protein level was found to be associated with TGF-β1-induced apoptosis and EMT (Fig. 1d). Increased YAP expression can clearly be induced by TGF-β1 treatment at a concentration of 1 ng/mL, but no further obvious increases were observed at higher concentrations (Fig. 1e). YAP is a transcriptional cofactor, and it functions primarily in the cell nucleus. Consistently, an increased YAP protein level was found in isolated nuclear fractions after TGF-β1 treatment (Fig. 1f). These results indicate that upregulation of YAP is associated with TGF-β1-induced cell apoptosis and EMT.

### YAP overexpression inhibits TGF-β1-induced apoptosis but not EMT

**Figure 2:**
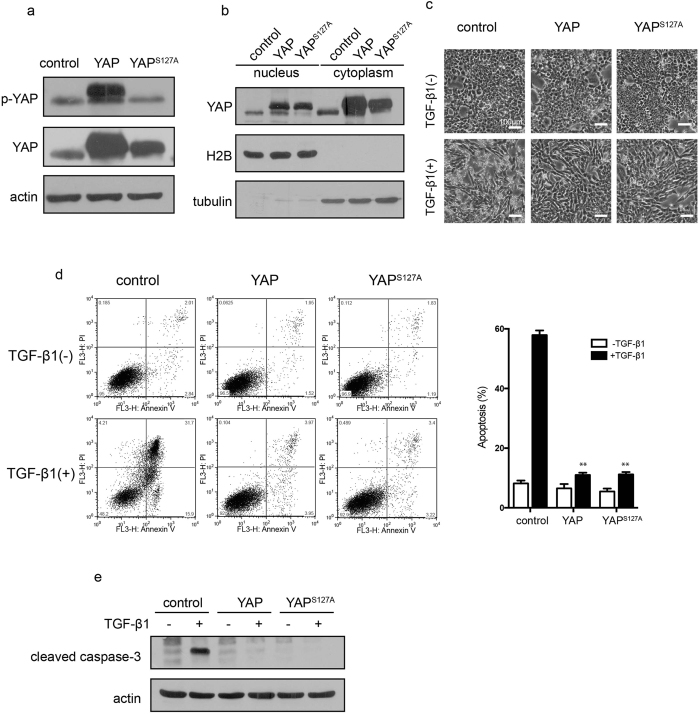
YAP inhibits TGF-β1-induced apoptosis. (**a**) Ectopic expression of YAP and YAP^S127A^ was detected by immunoblotting. (**b**) Nuclear expression of YAP was examined in YAP and YAP^S127A^ overexpressing cells. (**c**) Apoptotic cells and morphological changes were preliminarily observed by microscopic examination in YAP and YAP^S127A^ overexpressing cells 48 h after TGF-β1 treatment (10 ng/mL). Cells were imaged at 40× magnification. (**d**) The inhibition of TGF-β1-induced (10 ng/mL, 48 h) apoptosis by overexpression of YAP and YAP^S127A^ was detected by Annexin V/PI double staining. The statistic data shown are the means ± SD of three independent experiments. (**e**) The cleaved caspase-3 level was determined by immunoblotting in YAP and YAP^S127A^ overexpressing cells in response to TGF-β1 (10 ng/mL, 48 h).

As YAP was upregulated during TGF-β1-induced concomitant apoptosis and EMT, we explored the potential contribution of YAP to these two fundamentally different cell fates induced by TGF-β1. NMuMG cells overexpressing HA-tagged YAP and constitutively active YAP^S127A^ were constructed (Fig. 2a). As shown in Fig. 2b, overexpression of YAP was also associated with an increased nuclear localization. Strikingly, overexpression of either wild type or constitutively active YAP suppressed TGF-β1-induced cell death (Fig. 2c). Notably, YAP overexpression had no effect on morphological changes. The inhibitory effect of YAP on apoptosis was confirmed by Annexin V/PI double staining assay, which showed that forced expression of YAP remarkably reduced the number of both early and late apoptotic cells (Fig. 2d). YAP-mediated suppression of apoptosis was also shown by inhibition of TGF-β1-induced caspase-3 cleavage (Fig. 2e). In addition, in stable cell lines expressing two different levels of YAP, we observed that cell survival increased as the expression level of YAP increased (Supplementary Fig. S2a,b). Consistent with the cell morphology results, overexpression of YAP cannot induce EMT and has no effect on TGF-β1-induced EMT, as evidenced by the protein levels of EMT markers fibronectin and E-cadherin, although a relatively weak increase in N-cadherin was observed (Supplementary Fig. S2c). Moreover, YAP overexpression has no effect on E-cadherin level and F-actin arrangement induced by TGF-β1, as detected by immunofluorescent assays (Supplementary Fig. S2d and e). YAP overexpression also has no effect on the expression levels of Snail and PAI-1 genes (Supplementary Fig. S2f). The above results indicate that YAP has an anti-apoptotic function and its overexpression can substantially suppress TGF-β1-induced cell death. Although YAP has no effect on EMT directly, the number of surviving cells that undergo EMT can be enhanced as the expression level of YAP increases, strongly implying that the expression level of YAP may be involved in controlling the extent of either apoptosis or EMT in response to TGF-β1.

### Knockdown of YAP induces apoptosis

**Figure 3:**
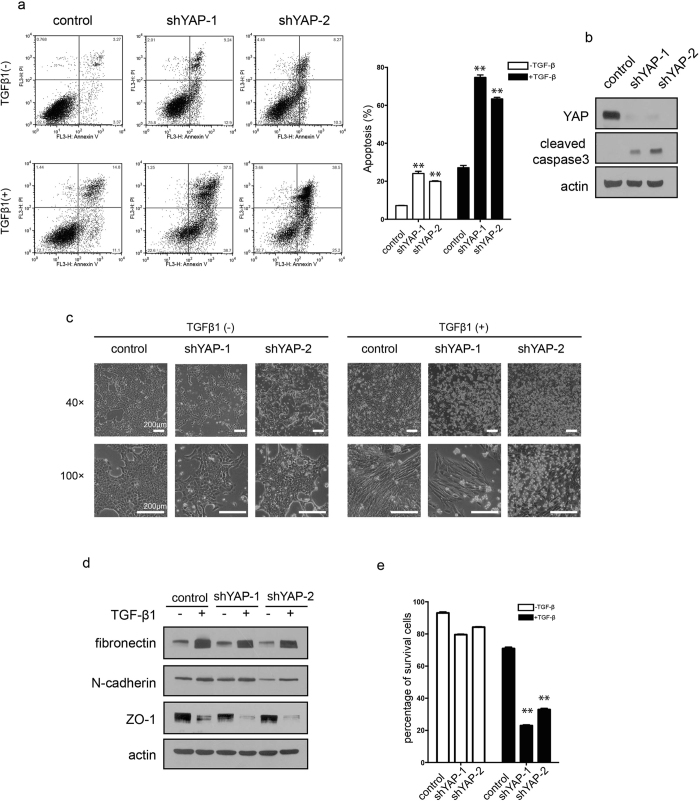
YAP knockdown promotes cell apoptosis in NMuMG cells. (**a**) Knockdown of YAP induced apoptosis spontaneously and promoted TGF-β1-induced apoptosis (5 ng/mL, 48 h), as detected by Annexin V/PI double staining in NMuMG cells. The representative images (left) and statistical data (right) were shown. The data are the means ± SD of three independent experiments. (**b**) Expression of cleaved-caspase 3 was determined by immunoblotting in YAP-knockdown cells. (**c**) Morphological changes in cells were examined in YAP-knockdown cells upon TGF-β1 treatment (5 ng/mL, 48 h). (**d**) The effect of YAP knockdown on EMT markers was examined upon TGF-β1 (5 ng/mL, 48 h) treatment. (Epithelial marker: ZO-1; Mesenchymal markers: fibronectin, N-cadherin). (**e**) Statistical analysis of the percentage of surviving cells (Annexin V negative/PI negative) in experiments conducted in (**a**). The data shown are the means ± SD of three independent experiments.

To further verify the role of YAP in the determination of cell fate of apoptosis versus EMT, we knocked down YAP by shRNA in NMuMG cells and then determined its possible effect. As examined by an Annexin V/PI double staining assay, YAP knockdown not only induced fundamental cell apoptosis at basal levels (Fig. 3a upper panel) but also enhanced TGF-β1-induced apoptotic cell death (Fig. 3a lower panel). The pro-apoptotic effect of YAP knockdown can be detected by the increased immunoblotting band of cleaved caspase-3 (Fig. 3b). These results further indicate that YAP is a critical survival factor for cells. Although YAP downregulation has no direct effect on TGF-β1-induced EMT, as shown by cell morphology (Fig. 3c) and EMT marker protein levels (Fig. 3d), it greatly reduced the numbers of surviving cells that could undergo EMT in response to TGF-β1. The FACS data analysis (Fig. 3e) showed that, in YAP-knockdown cells, TGF-β1 treatment reduced the percentage of viable cells (Annexin V/PI double negative) from approximately 70% to approximately 20%, while the viability of control cells went from 93% to 70%. The data suggest that, by directly inducing different degrees of apoptosis and thus indirectly influencing the percentage of viable cells which undergo EMT, YAP expression levels can exert a modulatory effect on the balance between cell apoptosis and EMT. In other words, significantly altered expression levels of YAP can break the balance of apoptosis and EMT in response to TGF-β1 stimulation.

**Figure 4:**
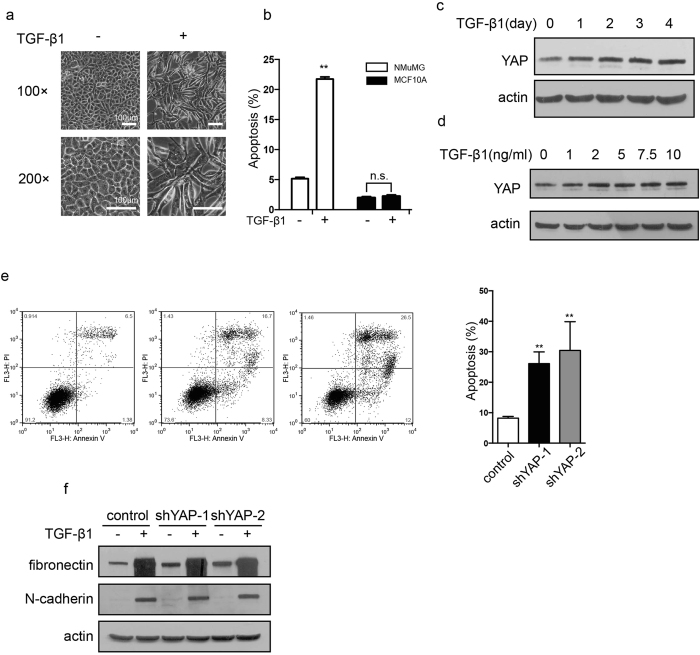
Induction of YAP expression and the YAP knockdown effect in MCF10A cells. (**a**) Morphological changes were examined in MCF10A cells after treatment with TGF-β1 (10 ng/mL) for 4 days. (**b**) The percentage of apoptotic cells were analyzed in NMuMG and MCF10A cell lines after treatment with TGF-β1 (10 ng/mL) for 48 h. The data shown are the means ± SD of three independent experiments. (**c**) The time effect of YAP expression was detected by immunoblotting after TGF-β1 (10 ng/mL) treatment for the indicated time in MCF10A cells. (**d**) The dosage effect of YAP expression was detected by immunoblotting after TGF-β1 treatment (48 h) with the indicated dosage in MCF10A cells. (**e**) Knockdown of YAP induced apoptosis spontaneously in MCF10A cells, as detected by Annexin V/PI staining. The representative images (left) and statistical data (right) were shown. The data are the means ± SD of three independent experiments. (**f**) Mesenchymal markers (fibronectin, N-cadherin) were detected by immunoblotting in YAP-knockdown cells after TGF-β1 (5 ng/mL, 48 h) treatment in MCF10A cells.

MCF10A is a human normal breast epithelial cell line that exhibits non-tumorigenic properties. Unlike NMuMG cells, MCF10A cells only underwent EMT (Fig. 4a), not apoptosis (Fig. 4b), in response to TGF-β1 stimulus. Similar to NMuMG cells, TGF-β1 treatment also slightly but obviously increased the expression level of YAP MCF10A cells, as shown in time- and dose-effect assays (Fig. 4c and d). To confirm that YAP is also important for cell survival in non-apoptotic cells upon TGF-β1 treatment, we knocked down YAP and determined its effect on cell viability. As shown in (Fig. 4e), YAP knockdown in MCF10A cells markedly induced spontaneous apoptosis. Interestingly, YAP knockdown also had no direct effect on TGF-β1-induced EMT in MCF10A cells (Fig. 4f), indicating that YAP is not a direct contributor to TGF-β1-induced EMT. YAP is critical for cell survival and can protect cells from TGF-β-induced cell death.

### Overexpression of YAP increases EGF receptor expression

**Figure 5:**
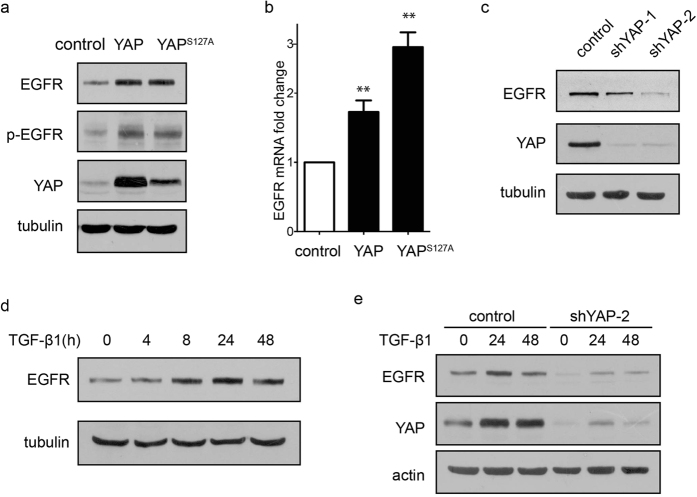
YAP regulates the expression level of EGFR. (**a**) EGFR and phospho-EGFR levels (Thr 1068) were detected in YAP and YAP^S127A^ overexpressing cells. (**b**) The relative mRNA level of EGFR was examined in YAP overexpressing cells. The data shown are the means ± SD of three independent experiments. (**c**) The expression level of EGFR was detected in YAP-knockdown cells. (**d**) The time-dependent protein level of EGFR was examined after TGF-β1 (10 ng/mL) treatment for the indicated time. (**e**) The expression of EGFR in control and YAP-knockdown cells after TGF-β1 treatment (10 ng/mL) was determined.

YAP and EGFR play similar important roles in cell survival and apoptosis-resistance. To understand the mechanism underlying the YAP-mediated suppression of TGF-β1-induced apoptosis, we examined whether the effect of YAP is associated with the EGFR level and its activation state. Indeed, overexpression of YAP increased the protein level and phosphorylated form of EGFR (Fig. 5a). An increased EGFR mRNA level was also observed in YAP-overexpressing cells (Fig. 5b). In contrast, knockdown of YAP inhibited EGFR expression (Fig. 5c). Since YAP is upregulated upon TGF-β1 treatment, we examined the effect of TGF-β1 on EGFR expression. Interestingly, TGF-β1 treatment also induced an increase in EGFR expression, which was time-dependent (Fig. 5d). Moreover, knock down of YAP abolished the TGF-β1-induced increase in EGFR (Fig. 5e). The data suggest that EGFR is a downstream molecule of YAP.

### EGFR is required for YAP-mediated anti-apoptotic function

**Figure 6:**
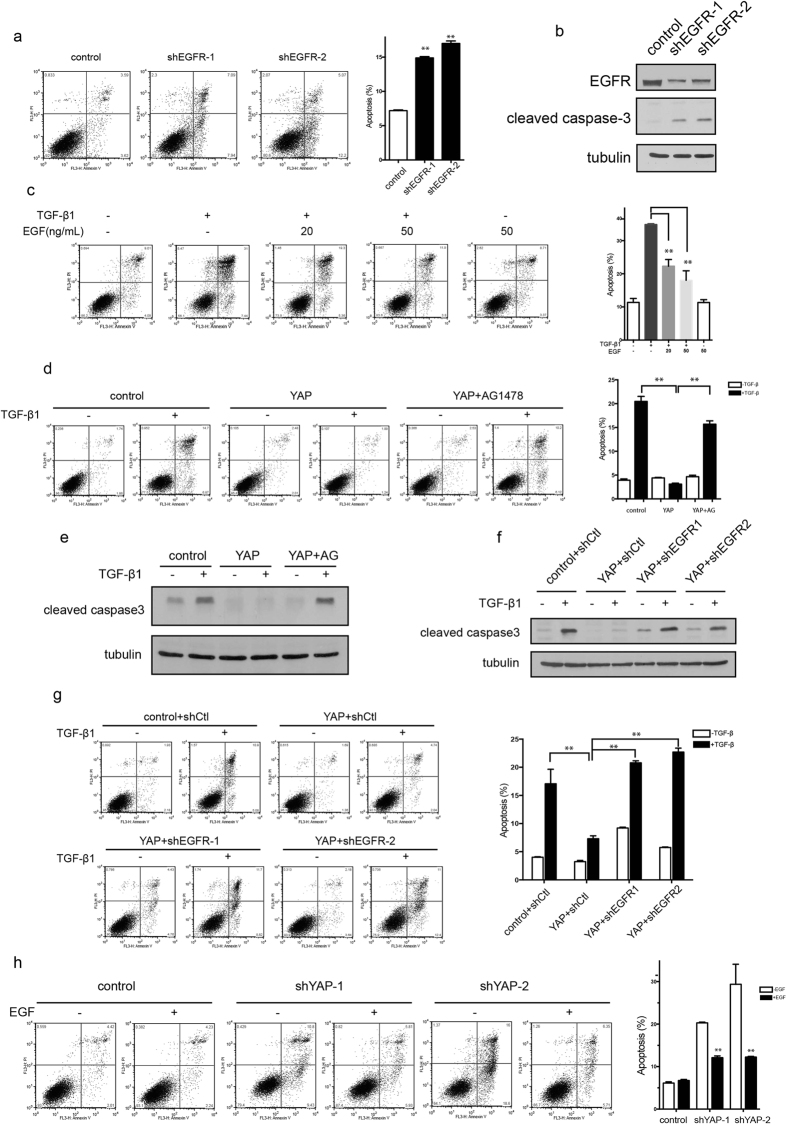
EGFR is required for YAP-mediated apoptosis inhibition. (**a**) EGFR knockdown induced apoptosis spontaneously, as detected by Annexin V/PI double staining. The statistic data shown are the means ± SD of three independent experiments. (**b**) The protein level of cleaved caspase-3 was examined in EGFR-knockdown cells. (**c**) Apoptotic cells were examined by Annexin V/PI staining after TGF-β1 (10 ng/mL, 48 h) treatment with or without EGF. Statistical data shown are the means ± SD of three independent experiments. (**d**) AG1478 inhibited YAP-mediated inhibition of TGF-β1-induced apoptosis. YAP overexpressing cells were treated with AG1478 (1 μM) 1 h before TGF-β1 (10 ng/mL) treatment, and the apoptotic cells were determined by Annexin V/PI double staining. The statistic data shown are the means ± SD of three independent experiments. (**e**) The protein level of cleaved caspase-3 was examined in YAP overexpressing cells treated with TGF-β1 (10 ng/mL) and AG1478 (1 μM). (**f**) The protein level of cleaved caspase-3 was examined in YAP overexpressing and EGFR-knockdown cells after TGF-β1 (10 ng/mL) treatment. (**g**) Apoptotic cells in YAP overexpressing and EGFR-knockdown cells were determined after TGF-β1 (10 ng/mL) treatment by Annexin V/PI double staining. The statistic data shown are the means ± SD of three independent experiments. (**h**) Apoptotic cells were examined in YAP-knockdown cells with or without EGF (50 ng/mL) by Annexin V/PI double staining. The statistic data shown are the means ± SD of three independent experiments.

EGFR is an important survival factor in cells, and its knockdown in NMuMG cells caused marked apoptosis, as shown by Annexin V/PI double staining (Fig. 6a) and increased the level of cleaved caspase-3 (Fig. 6b). To investigate whether EGFR is involved in the YAP-mediated effect, cells were treated with TGF-β1 in the presence of EGF. The addition of exogenous EGF suppressed TGF-β1-induced apoptosis (Fig. 6c and Supplementary Fig. S3). Inhibition of EGFR by AG1478 abolished the apoptotic resistance effect mediated by YAP, as assessed by Annexin V/PI double staining (Fig. 6d) and cleaved caspase-3 level (Fig. 6e). These results suggest that EGFR is implicated in YAP-mediated anti-apoptotic function in response to TGF-β1. In addition, knockdown of EGFR in YAP overexpressing cells (Supplementary Fig. S4) also repressed the YAP-mediated suppression of TGF-β1-induced apoptosis, as shown by the level of cleaved caspase-3 (Fig. 6f) and FACS (Fig. 6g). Moreover, EGF rescued the apoptosis induced by YAP knockdown (Fig. 6h). These data suggest that EGFR accounts, at least partly, for the suppression of TGF-β1-induced apoptosis by YAP in NMuMG cells.

## Discussion

TGF-β1 signaling exerts multiple effects depending on different cellular contexts and environments. In non-cancer cells, such as NMuMG and AML12 cells, TGF-β1 can simultaneously induce apoptosis and EMT. The balance between apoptosis and EMT is critical to embryonic development, organogenesis, tissue repair, and regenerative processes. From the view of tumor transformation, the apoptotic effect has often been termed a tumor-suppressing factor, while EMT has been known as a tumor-promoting factor. Cancer cells overcome tumor suppressive barriers by harboring cell migration and metastatic abilities. Exploring the molecules that regulate apoptosis and EMT induced by TGF-β1 and the balance of these two fates is critical for understanding both the physiological and the pathological processes. In the present study, we showed that simultaneous apoptosis and EMT in NMuMG cells could be strongly induced by TGF-β1 treatment. YAP overexpression enabled non-transformed NMuMG cells to overcome TGF-β1 mediated apoptosis; while in YAP-knockdown cells, a large portion of cells underwent spontaneous apoptosis and increased apoptotic response to TGF-β1 stimulation. Our results indicate that YAP is vital in regulating the cell survival rate in response to TGF-β1. Although we found that YAP did not influence the induction of EMT directly, the expression of YAP controlled the numbers of cells that underwent EMT by regulating the survival rate of cells. Thus, YAP is vital for the balance of cell fate in apoptosis and EMT, which may be critical during embryonic development and in maintaining tissue homeostasis in living bodies.

In the induction of simultaneous apoptosis and EMT by TGF-β1, although a portion of cells died, another portion of cells survived and underwent EMT. This fact suggests that both pro-apoptotic and anti-apoptotic molecules are involved in the TGF-β1-mediated effect. The balance or the extent of apoptosis or EMT may also be influenced by the levels of pro-apoptotic and anti-apoptotic molecules. It is worth noting that the samples collected for examining YAP and EGFR expression levels were mainly obtained from intact viable cells because apoptotic cells could not be collected effectively due to floating and degradation. In this case, it can be concluded that increased YAP expression was primarily from cells undergoing EMT. Consistent with our results, it has been reported that several classical anti-apoptotic molecules, such as PI3K-AKT, ERK, and c-Src, are activated in response to TGF-β1 in NMuMG cells and hepatocytes, which resist TGF-β1 mediated apoptosis^35^,^36^,^37^,^38^,^39^. In addition, MSK1^40^, Dlx2^41^, and lncRNA-smad7^42^ have been shown to have an effect of anti-TGF-β1-induced apoptosis. However, their expression levels are also elevated during TGF-β1-induced apoptosis or EMT. Thus, the levels of anti-apoptotic molecules are essential for a portion of cells to survive and be resistant to the apoptotic effect of TGF-β1 stimulation. YAP downregulation markedly reduced the cell survival rate and a correspondingly decreased proportion of cells underwent EMT, suggesting that sufficient levels of YAP are necessary for cells to be viable and able to undergo EMT. Although TGF-β1-induced simultaneous EMT and apoptosis have been shown to be cell cycle-dependent events^14^, it still remained unclear what molecular events are linked with different cell cycle phases.

Interestingly, a decade ago, Snail has been shown to block cell cycle and confers cell resistance to death in MDCK cells 1^43^. Several years after that, it has also been shown that overexpression of snail induced EMT and suppressed TGF-β1-induced apoptosis in mouse hepatocytes^44^. These reports strongly support our new finding about the regulatable balance of apoptosis and EMT. In both cases, the anti-apoptotic effect of Snail are most likely associated with or dependent on its EMT inducing effect, because the following two reasons. Firstly, in both MDCK and hepatocytes, EMT can be promoted by TGF-β1 treatment or by Snail overexpression. Moreover, both apoptosis and EMT are the two strong simultaneous responses of hepatocytes to TGF-β1 treatment. Secondly, the induction of EMT generally presupposes cell growth arrest or decelerated proliferation, which can be more prominent in TGF-β1-induced EMT in non-cancer cells. It has been recently reported that TGF-β treatment in pancreatic ductal adenocarcinoma cells induces a lethal EMT that leads to apoptosis^45^. Due to the lack of a basic comparison of the time-effect between the apoptosis and EMT induced by TGF-β, the conclusion that EMT preceded apoptosis cannot be made. The relevant conclusion was also not backed up by other assays important for detecting the early apoptotic response of cells to TGF-β. Besides, the parallel assay showing the Snail overexpression induced EMT was not provided to support the assumed apoptosis-inducing effect of Snail. It is important to note that, in TGF-β-induced concomitant apoptosis and EMT in culture dishes, the EMT only temporarily, but not permanently, enabled cell to escape apoptotic inducing effect of TGF-β.

YAP overexpression alone was able to induce EMT in MCF10A cells (Supplementary Fig. S5), which is consistent with published reports^31^,^46^. Nevertheless, both overexpression and downregulation of YAP showed no obvious effects on TGF-β1-induced EMT in NMuMG cells. These results suggest that the function of YAP in EMT is cell type-dependent. In addition, the finding that knockdown of YAP in MCF10A cells cannot inhibit TGF-β1-induced EMT indicates that YAP is not required for the induction. In spite of the fact that YAP has no direct effect on TGF-β1-induced EMT, YAP levels are important for cell viability, which indirectly influences the number of cells undergoing EMT. Thus, during the processes of simultaneous apoptosis and EMT stimulated by TGF-β1, YAP plays a modulatory effect in determining the extent or trend of cell death versus EMT.

YAP is primarily considered an oncogenic molecule and high levels of YAP predict poor prognosis in primary tumors^27^. YAP expression levels in various cancer types are higher than in normal tissues, which may provide an explanation for the fact that cancer cells often escape from the apoptotic effect and acquire the pro-metastasis effect of TGF-β1. Our findings that YAP functions as a potent suppressor of apoptosis and thus can indirectly modulate the concomitant apoptosis and EMT induced by TGF-β1 imply a novel effect of YAP in relevant patho-physiological processes.

Epidermal growth factor receptor (EGFR) plays important roles in anti-apoptosis and solid tumor progression. The functions of EGFR and YAP in cell survival and tumor promotion are similar; thus, we examined whether EGFR could be regulated by YAP. TGF-β1 increased the expression of EGFR, and knockdown of YAP inhibited the upregulation of EGFR upon TGF-β1 stimulation. Inhibition of EGFR activity or knockdown of EGFR in YAP-overexpressing cells suggests that EGFR is involved in YAP-mediated cell survival. YAP has been shown to be able to transcriptionally upregulate EGFR in esophageal cancer tissues through TEAD binding sites^47^; in ErbB2/EGFR-transgenic mice, increased YAP level in nuclei of mammary glands was observed^48^. YAP has also been shown to activate the EGFR by increasing the expression of an EGFR ligand, amphiregulin^49^,^50^. These evidences suggest a mutual interaction between the Hippo and EGFR signaling pathways. We did not observe the induction of exogenous EGFR promoter activity by forced YAP expression (Supplementary Fig. S6). It is possible that the upregulation of EGFR by YAP is not through direct binding of YAP to the EGFR promoter in normal mammary gland cells. However, further exploration of the mechanism by which YAP regulates EGFR expression is important on this aspect. As it has been shown recently that most YAP/TAZ-bound regions coincide with enhancer elements^51^, one of the possibilities is that EGFR expression is regulated by YAP through an enhancer region.

**Figure 7:**
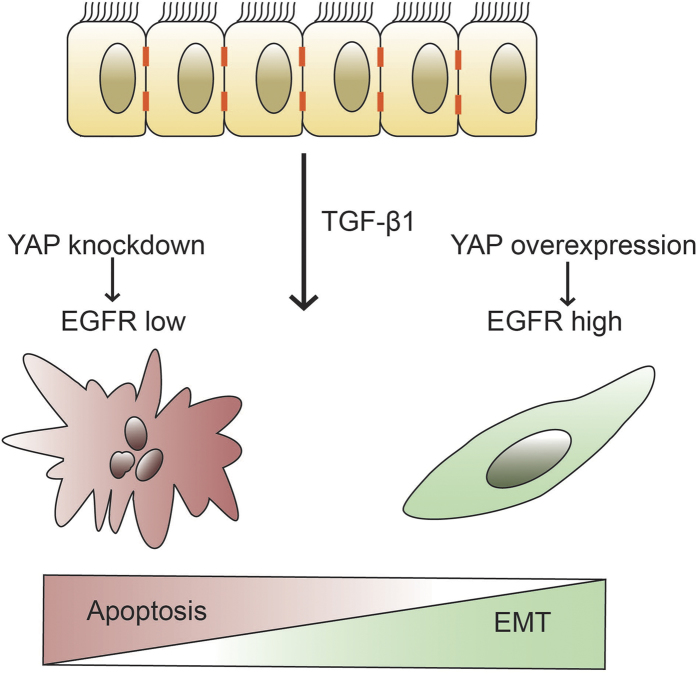
An illustration of the YAP effects on simultaneous apoptosis and EMT mediated by TGF-β1. TGF-β1 concomitantly induces apoptosis and EMT of different portions of cells. Overexpression of YAP elevated EGFR levels, which increased cell survival and protected cells from apoptosis, leading to a marked increase in the number of cells undergoing EMT. YAP knockdown induced spontaneous apoptosis and enhanced apoptotic response of cells to TGF-β1, causing the balance between apoptosis and EMT to be tilted toward apoptosis, which also reduced the cell survival rate and thus the number of cells that underwent EMT.

To briefly summarize, the presented results demonstrated that YAP plays a critical role in modulating cell fate in response to TGF-β1-mediated apoptosis and EMT (Fig. 7). Overexpression of YAP suppressed apoptosis and increased the number of cells undergoing EMT. In contrast, downregulation of YAP reduced cell viability, leading to significant spontaneous apoptosis and an enhanced apoptotic response to TGF-β1 treatment, which also led to a pronounced decrease in the proportion of cells that underwent EMT. Further investigation found that YAP overexpression is associated with increased EGFR expression and activation. Inhibition of EGFR expression or activation blocked the anti-apoptotic effect of YAP, suggesting that the modulating effect of YAP on TGF-β1-induced apoptosis and EMT requires EGFR signaling. This study provides an insight into the regulation of cell fate through apoptosis and EMT induced by TGF-β1.

## Methods

### Cells and cell culture

NMuMG (non-transformed murine mammary gland), MCF10-A and HEK293T cells were purchased from the American Type Culture Collection (ATCC, Manassas, VA, USA). NMuMG cells were cultured in Dulbecco’s modified Eagle’s medium (Gibco, Grand Island, NY, USA) with 10% fetal bovine serum (Gibco), plus 10 μg/mL insulin (Sigma, St Louis, MO, USA). MCF10A cells were cultured in DMEM/F12 supplemented with 5% horse serum, 10 μg/mL insulin, 20 ng/mL EGF, 100 ng/mL cholera toxin and 0.5 μg/mL hydrocortisone^52^. HEK293T cells were cultured in DMEM plus 10% FBS. The cells were incubated at 37 °C in a humidified atmosphere with 5% CO_2_. Experiments were performed when cells reached 40–50% confluence.

### Reagents and plasmids

Human recombinant TGF-β1 was purchased from ChemiCon (Millipore, Billerica, MA, USA). Antibodies against Yap, phospho-Yap (Ser 127), H2B, caspase-3, cleaved caspase-3, and phospho-EGFR (Thr 1068) were purchased from Cell Signaling (Danvers, MA, USA). Antibodies against E-cadherin and N-cadherin were obtained from BD Biosciences (Franklin Lakes, NJ, USA). Antibody against ZO-1 was obtained from Invitrogen (Thermo-Fisher, Rockford, IL, USA). The antibodies against tubulin and fibronectin were purchased from Sigma (St Louis, MO, USA). EGF was purchased from Peprotech (Rocky Hill, NJ, USA) and AG1478 from Selleck (Shanghai, China). Cells were transfected using a lenti-virus system. Plasmids pCDH-CMV-MCSEF1-Puro, psPAX2, pMD2.G and pLKO.1-TRC were purchased from Addgene (Cambridge, MA, USA). The shRNAs were constructed in pLKO.1 with a target sequence as follows:

Mouse Yap-1: ACTTGGAGGCGCTCTTCAATG,

Mouse Yap-2: TGAGAACAATGACAACCAATA,

Human Yap-1: GACATCTTCTGGTCAGAGA

Human Yap-2: CCACCAAGCTAGATAAAGAA

Mouse EGFR-1: GGGAAATGCTCTTTATGAA

Mouse EGFR-2: AACTTACAGGAAATCCTGATT

### Immunoblotting

Cells were lysed in lysis buffer (50 mM HEPES, 5 mM EDTA, 50 mM NaCl, 1% Triton X-100, 50 mM NaF, 10 mM Na_4_P_2_O_7_ · 10 H_2_O, protease inhibitors, pH 7.4). Proteins were separated by SDS-PAGE according to molecular weight and transferred onto nitrocellulose membranes (PALL, Port Washington, New York, USA). Membranes were blocked with 5% skim milk and were incubated with the appropriate primary antibodies diluted 1:2500 and then secondary antibodies conjugated with HRP. Protein bands were visualized by an ECL reagent.

### Immunofluorescent staining

Immunofluorescent staining was performed as previously described^53^.

### RNA isolation and real-time PCR

Total RNA was extracted by TRIzol reagent (Tiangen, Beijing, China) and phenol/chloroform methods were performed according to the manufacturer’s instructions. Briefly, 1 μg of RNA was used for reverse transcription by ReverTra Ace (Toyobo, Osaka, Japan). cDNAs were subjected to real-time PCR with a 7500 Fast Real-Time PCR system (ABI, Thermo-Fisher) using Power SYBR Green PCR Master Mix (CWBio, Beijing, China). Specific primer sequences are as follows:

mEGFR-f: TGAAGAGGACATGGAGGATG

mEGFR-r: CACTCAGAGAACTCAAGAGG

mSnail-f: GTGTGTGGAGTTCACCTTCC

mSnail-r: AGGAGAGAGTCCCAGATGAG

mPAI1-f: TTCCACAAGTCTGATGGCAG

mPAI1-r: GGTGGTGAACTCAGTGTAGT

### Annexin V-FTIC/PI double staining assay

Cells were trypsinized and washed with PBS twice to remove residual EDTA. Cells were stained with Annexin V-FITC/PI for 10 min in binding buffer (0.01 M HEPES, 0.14 M NaCl; 2.5 mM CaCl_2_, pH 7.4). Stained cells were detected by flow cytometry (FACS Calibur, Becton Dickinson, Franklin Lakes, NJ, USA). To gate the apoptotic region, single-stained cells were used as negative controls. The percentage of apoptotic cells were quantified by the sum of Annexin V^+^PI^−^ and Annexin V^+^PI^+^ cells, as shown in the lower right and upper right quadrants of FACS assay.

### Stable transfection

Lenti-viruses were packaged in HEK293T cells by calcium phosphate transfection with plasmid. Viruses were collected 36 h after transfection and filtered through 0.45-μm filters. The host cells were infected by the collected viruses plus 10 μg/mL polybrene and selected with puromycin.

### Nuclear isolation

Cells were harvested and washed with PBS and then suspended in hypotonic buffer (10 mM HEPES, 1.5 mM MgCl_2_, 10 mM KCl, 0.5 mM DTT, protease inhibitors, pH 7.9) for 10 min. Cells were scraped and mixed with 10% NP-40 and then centrifuged. The supernatant contained cytosolic components and the precipitate contained the nuclei. The nuclei were resuspended in hypertonic buffer (20 mM HEPES, 1.5 mM MgCl_2_, 420 mM NaCl, 0.2 mM EDTA and 25% glycerol, protease inhibitors, pH 7.9).

### Luciferase reporter assay

A mouse EGFR promoter was constructed in a pGL3-Basic vector and co-transfected into NMuMG cells with a pRL-TK Vector by Lipofectamine 2000 (Invitrogen, Thermo-Fisher, Rockford, IL, USA). Measurements of luciferase activity were performed according to the manufacturer’s instructions.

### Statistical analyses

Quantitative data from at least three experiments were compared and normalized to control cells. For experiments with two groups, statistical significance was determined by a Student’s t-test. For experiments involving more than two groups, statistical analyses were performed by ANOVA followed by pairwise comparison. A P-value < 0.05 was considered statistically significant. *P < 0.05; **P < 0.01; n.s., non significant.

## Additional Information

**How to cite this article:** Liu, Y. *et al*. YAP modulates TGF-b1-induced simultaneous apoptosis and EMT through upregulation of the EGF receptor. *Sci. Rep.* **7**, 45523; doi: 10.1038/srep45523 (2017).

**Publisher's note:** Springer Nature remains neutral with regard to jurisdictional claims in published maps and institutional affiliations.

## Supplementary Material

Supplementary Information
