# Supplementary material for: YAP modulates TGF-β1-induced simultaneous apoptosis and EMT through upregulation of the EGF receptor
Source: Sci Rep. 2017 Apr 20;7:45523. doi: 10.1038/srep45523 (PMC5397873; doi:10.1038/srep45523)
Supplement: Supplementary Information [file srep45523-s1.pdf]

YAP modulates TGF-β1-induced simultaneous apoptosis and EMT through upregulation of the EGF receptor

Yi Liu, Kai He, Ying Hu, Xiaojie Guo, Dongmei Wang, Weiwei Shi, Jingsong Li and Jianguo Song*

State Key Laboratory of Cell Biology, Innovation Center for Cell Signaling Network, Institute of Biochemistry and Cell Biology, Shanghai Institutes for Biological Sciences, Chinese Academy of Sciences. Shanghai 200031, China

*Correspondence to: Jianguo Song

Phone& Fax: 86-21-54921167; E-mail: jgsong@sibcb.ac.cn

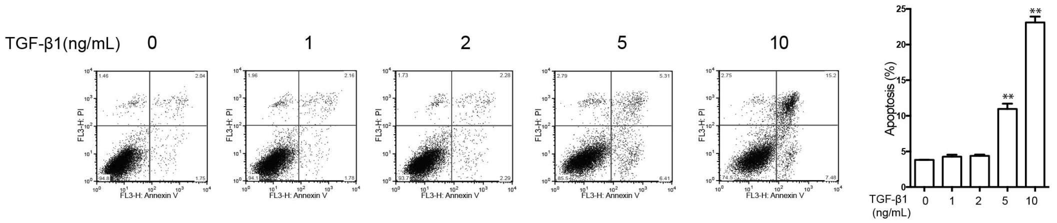

Supplementary Figure S1

The dosage effect of TGF-β1 on cell apoptosis

NMuMG cells were treated with TGF-β1 at indicated concentrations for 48 h, and the apoptotic cells were detected by Annexin V/PI double staining. The representative images (left) and statistical data (right) were shown. The data are the means ± SD of three independent experiments.

Supplementary Figure 2

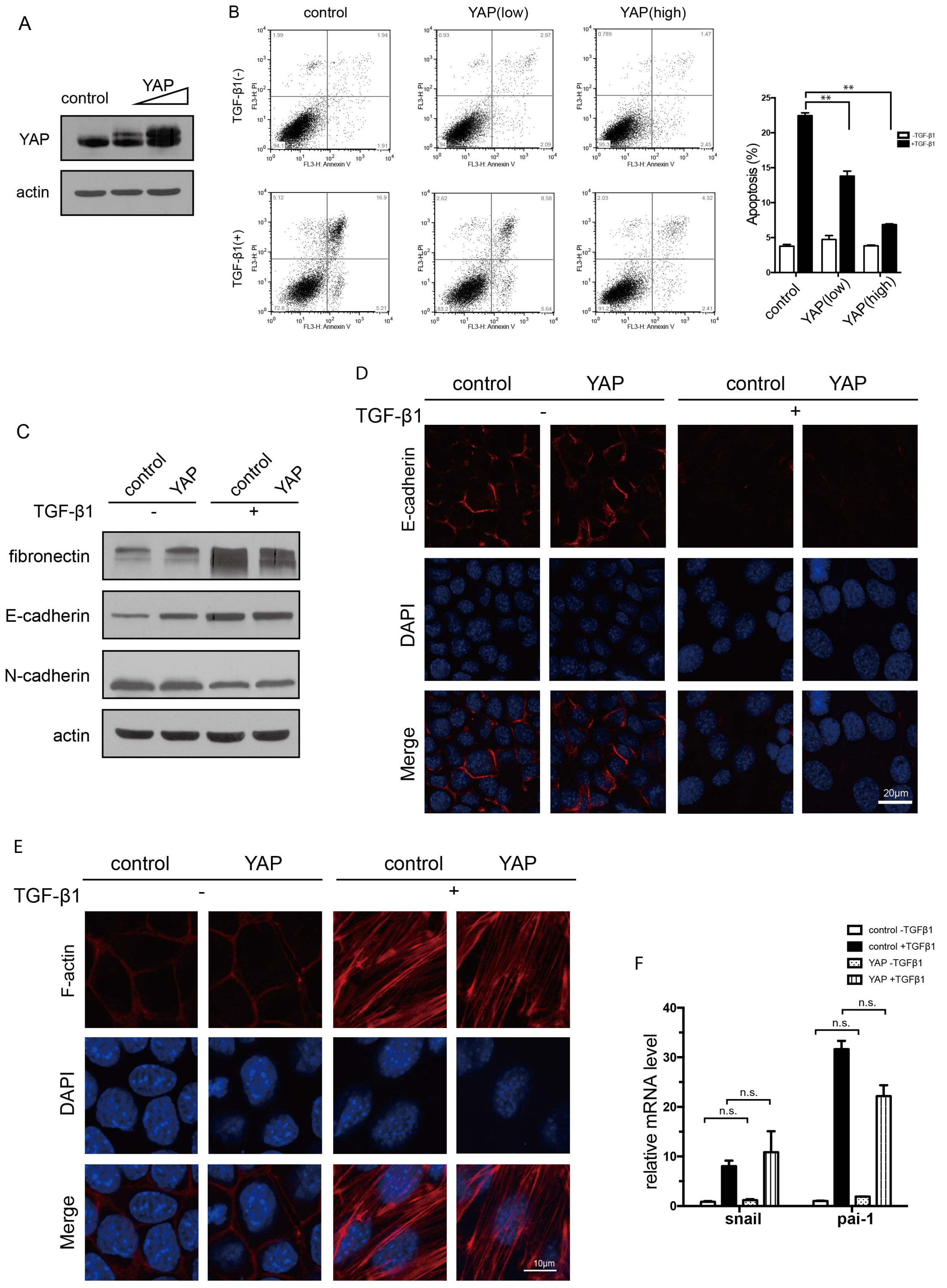

Supplementary Figure S2

The effect of YAP overexpression on TGF-β1-induced apoptosis and EMT

(a). Two different amounts of YAP were transfected into NMuMG cells and detected by immunoblotting. (b). The inhibitory effect of YAP on apoptosis

induced by TGF-β1 (10 ng/mL, 48 h) was determined by Annexin V/PI double staining. The statistic data shown are the means ± SD of three independent experiments. (c). The EMT marker protein levels were detected in YAP and YAPS127A overexpressing cells with and without TGF-β1 treatment. (d and e). Immunostaining of E-cadherin and F-actin in control and YAP overexpression cells treated with 5 ng/ml TGF-β1 for 48 h. Nuclei were stained with DAPI. Representative results from three independent experiments are shown. (f) The mRNA level of Snail and PAI-1 was examined in YAP overexpressing cells upon TGF-β1 (5 ng/mL, 48 h) treatments. The data shown are the means ± SD of three independent experiments.

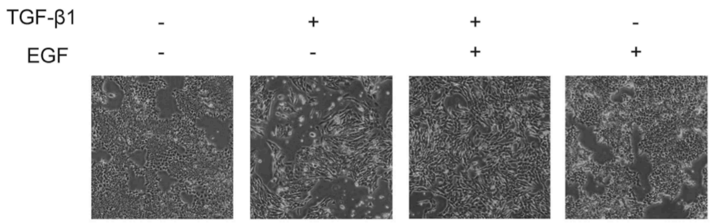

Supplementary Figure S3

The effect of EGF on TGF-β1-induced apoptosis and EMT

The morphological changes in NMuMG cells were detected after TGF-β1 treatment (10 ng/mL) with or without EGF (50 ng/mL).

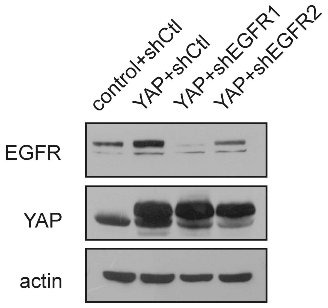

Supplementary Figure S4

EGFR knockdown in YAP overexpressing cells

EGFR KD was achieved by transfection of a shEGFR plasmid into YAP overexpressing cells. The knockdown effect was determined by immunoblotting with an anti-EGFR antibody.

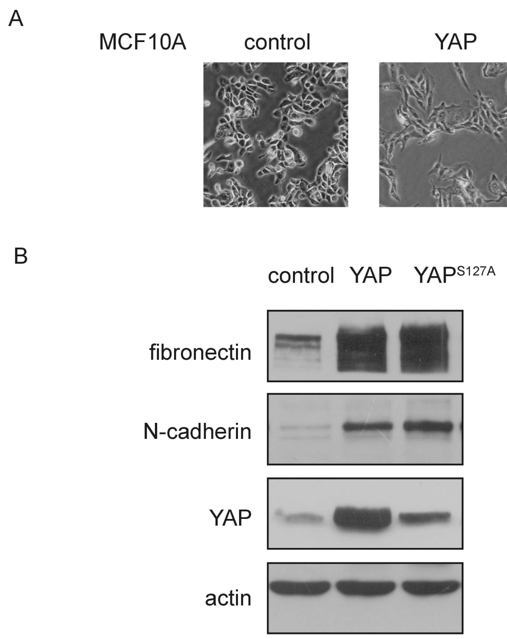

Supplementary Figure S5

The effect of YAP overexpression on EMT in MCF10A

(a). The morphological changes in YAP-overexpressing MCF10A cells and control cells. (b). The effect of YAP overexpression on EMT markers was examined.

Supplementary Figure S6

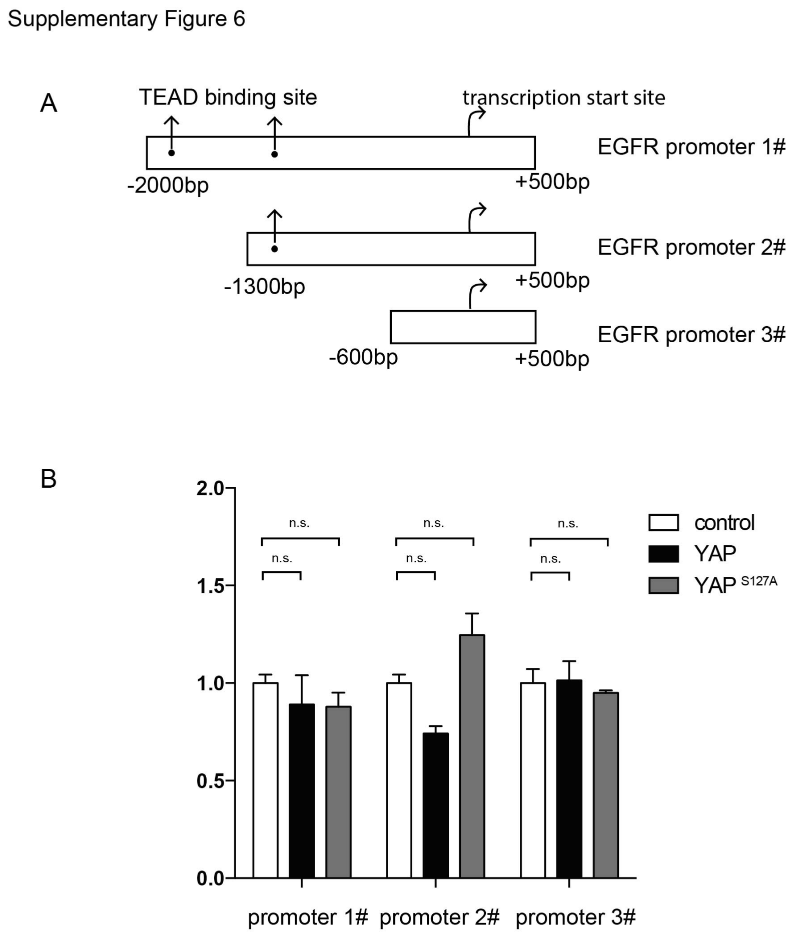

The effect of YAP on EGFR promoter transcription activity

(a). Schematic graph of EGFR promoter constructs. Truncated promoters that did or did not contain the predicted TEAD binding site were designed. (b). Different lengths of promoters were transfected into YAP-overexpressing or control cells. The luciferase activity was assessed to reflect the promoter transcriptional activity.
